# Obstructive Giant Inflammatory Polyp of the Colon in Ulcerative Colitis

**DOI:** 10.7759/cureus.45535

**Published:** 2023-09-19

**Authors:** Resheed Alkhiari, Atheer M Alharbi, Hala Albadrani, Jolan S Alsaud, Khaled Alkhiari

**Affiliations:** 1 College of Medicine, Qassim University, Qassim, SAU; 2 Department of Medicine, King Fahad Medical City, Riyadh, SAU

**Keywords:** inflammatory bowel disease, inflammatory polyposis, pseudo-polyps, ulcerative colitis, giant inflammatory polyp

## Abstract

Inflammatory polyps, also known as pseudo-polyps, are a common benign condition affecting 10-20% of patients with inflammatory bowel disease. Chronic, repeated inflammation and ulceration associated with healing lead to the formation of polyp-like structures in the colon. Although there are no common symptoms accompanying these pseudo-polyps, they can present with anemia, weight loss, diarrhea, intussusception, palpable mass, abdominal pain, discomfort, and melena, not to mention bowel obstruction that happens infrequently. Finally, it is important to recognize giant inflammatory polyps as they may occasionally be mistaken for colon cancer, leading to unnecessary surgical interventions. We present the case of a 38-year-old woman who was diagnosed with ulcerative colitis 10 years back, treated with oral mesalamine for five years, and had no follow-up after this period. She came to our clinic complaining of recurrent obstructive symptoms for a few months. Examination shows tenderness in the left lower quadrant with normal vital signs and bowel sounds.

## Introduction

Inflammatory polyps or pseudo-polyps are a well-known complication of inflammatory bowel disease. They develop in 10-20% of patients with the disease [1]. Pseudo-polyps may appear after periods of chronic inflammation and ulceration but do so frequently with inflammatory bowel disease, in particular, ulcerative colitis (UC) [2]. According to the suggested etiology, pseudo-polyps develop from residual islands of mucosa that remain after the surrounding, severely inflamed mucosa becomes ulcerated. This process of regeneration and healing leaves inflamed colonic mucosa in a polypoid pattern [3-5]. Rarely, these polyps can grow larger than 1.5 cm in dimension, at which point they are termed giant inflammatory polyps (GIPs) [6].

Pseudo-polyps usually cause no symptoms and are discovered incidentally during colonoscopy. However, they may manifest clinically with various symptoms, including weight loss, melena, anemia, diarrhea, cramping abdominal pain, palpable mass, and intussusception. In very rare cases, they show obstructive symptoms [7-8].

Their significance stems from the fact that, among these individuals with UC who are at higher risk for colonic malignancy, GIPs are frequently mistaken clinically and radiologically for carcinomas, potentially leading to unnecessary surgery.

## Case presentation

A 38-year-old woman was evaluated at our department with a 10-year history of UC. She was initially diagnosed with the disease based on symptoms and a colonoscopy with biopsies, which confirmed chronic ulcerative pancolitis. Thereafter, she was placed on oral mesalamine and remained in clinical remission for 5 years. However, she was lost to follow-up and has not been on treatment since. This time, she was seen and evaluated regarding obstructive symptoms including bloating, constipation, and lower abdominal pain associated with weight loss. These symptoms had been recurrent for a few months. At presentation, she was experiencing mild abdominal pain.

**Figure 1:**
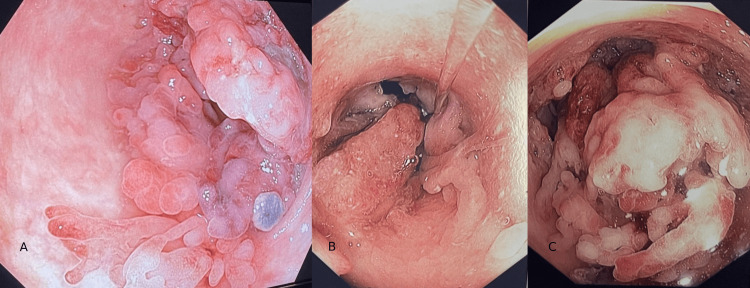
Endoscopic view of a large inflammatory polyp causing obstruction of the colonic lumen.

On physical examination, the patient was afebrile (36.5◦C), and her abdomen was tender in the left lower quadrant with no palpable masses or signs of peritonitis. The bowel sounds were normal. Laboratory investigations showed mild microcytic hypochromic anemia (MCV = 69 femtoliters, MCHC = 25 grams/deciliter, Hb = 11.2 grams/deciliters), normal white blood cell counts, and mildly elevated C-reactive protein at 7 mg/dL. A colonoscopy was then performed as an outpatient, and quiescent pancolitis with a large near-obstructing mass approximately 5 cm in size at the descending colon was demonstrated (Figures 1A-1C). Using a pediatric colonoscope (Olympus 190 series, Olympus America Inc., Center Valley, PA), the whole colon to the terminal ileum was examined. The initial impression was of a malignant polyp, and multiple deep and segmental biopsies were obtained from the lesion. Histopathological examination showed pseudo-polyps with no sign of dysplasia. 

**Figure 2:**
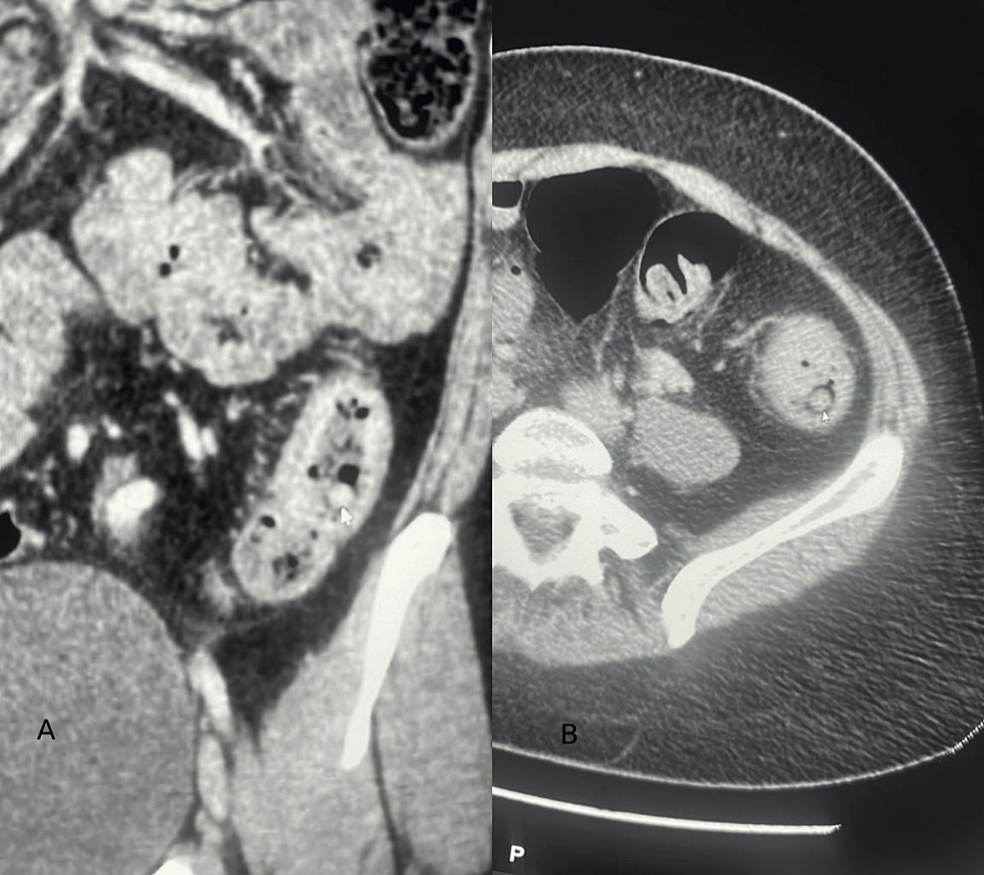
CT scan showing a focal circumferential proximal sigmoid wall thickening spanning 5.2 cm with a maximum thickness of 0.8 cm. It is associated with surrounding fat stranding and congested paracolic vessels.

An abdominal computed tomography scan was then performed to evaluate any extramural extension of the lesion. The scan revealed a large semi-obstructive mass at the descending colon (Figures 2A-2B).

**Figure 3:**
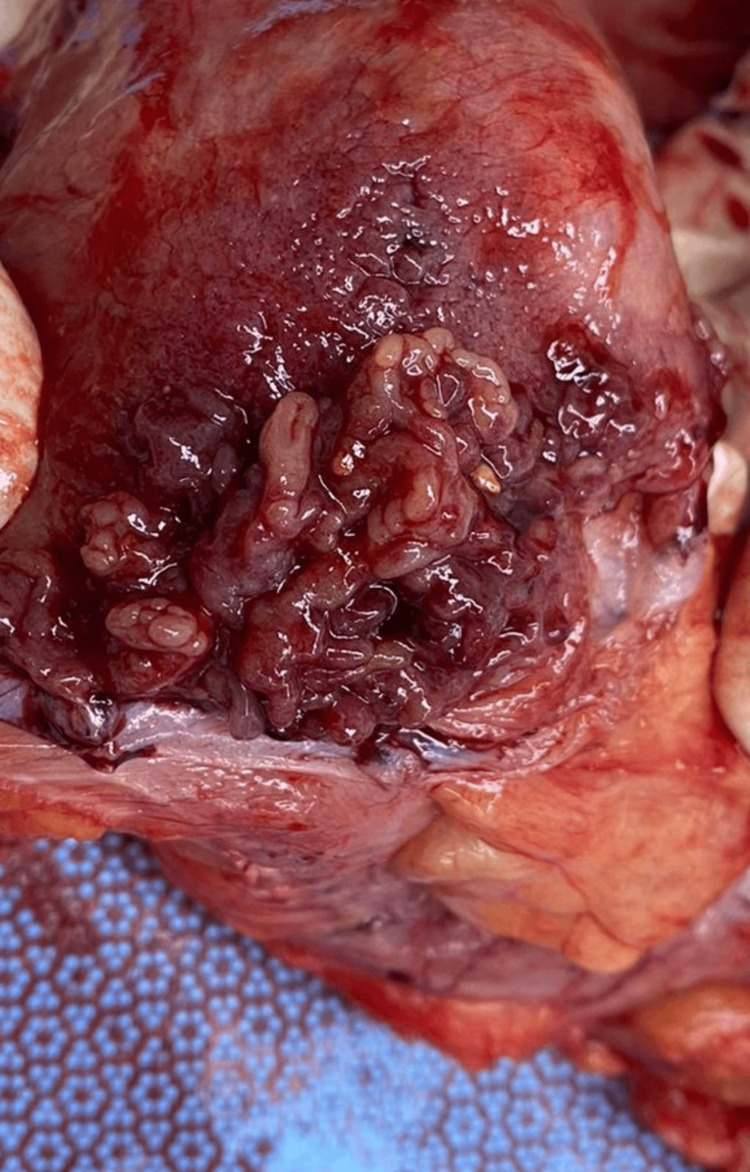
Gross examination of the resected colon with obstructing polyps.

Although biopsies suggested inflammatory polyps, a large mass underlying dysplastic and malignant alterations could not be ruled out. In most cases, most biopsies in a large mass can be superficial. For that reason, a multidisciplinary team, including a colorectal surgeon, discussed the case in detail regarding the best care for the patient. The recommended plan was shared with the patient, who agreed to undergo a total colectomy. She then underwent a total colectomy with ileorectal anastomosis, and her recovery was uneventful (Figure 3). The patient was seen in follow-up with good recovery and total resolution of previous symptoms.

## Discussion

GIPs are associated with inflammatory bowel disease with chronic pancolitis. They are extremely rare and can result in large bowel obstruction. The first report was in 1965 when Dr. Goldgraber described a localized recurrent giant pseudo-polyposis [9]. 

In the present case report, the patient had chronic ulcerative pancolitis for more than 10 years and was not on therapy for the last five years. Her symptoms were bloating, constipation, and weight loss associated with lower abdominal pain rather than acute flare, which usually is accompanied by diarrhea. Further evaluation revealed a large obstructing colonic mass in the form of a pseudo-polyp approximately 5 cm in size, which was managed with surgical resection.

GPP typically presents with symptoms of inflammatory bowel disease flares, such as abdominal pain and diarrhea, but rarely presents with obstructive symptoms. In a systematic review by Maggs et al., 78 patients with GPP were studied. Of them, 15% had complications related to obstruction and sub-obstruction, and 3% had mechanical intussusception due to the GPP's large size [10]. 

Ooi et al. showed that the median time from diagnosis to developing GIPs for UC and Crohn's disease is five and six years, respectively [11]. In contrast, Yada et al. observed a wide variation in time, from three to 276 months, between the diagnosis of GIPs and the initial diagnosis of inflammatory bowel disease, with possible early development of giant pseudo-polyps [12].

Pseudo-inflammatory polyps are an independent indicator of a higher risk of malignancy. Multivariate analysis revealed that PIPs are associated with a twofold increase in the risk of malignancy (OR 2.29; 95%CI 1.28-4.11) and a four-fold increase in colonic strictures (OR 4.62; 95%CI 1.03-20.8) when 68 cases of colorectal cancer complicating UC were compared with 136 matched controls [13].

GIPs, however, are viewed as a benign phenomenon. Only one instance of occult carcinoma developing in a patient with localized GIP has been documented [13]. Even if a GIP is appropriately diagnosed using radiography, endoscopy, and histopathology, a carcinoma cannot be ruled out [10].

Different modalities are used for the treatment of PPs, including medical, endoscopic, and surgical. Regression of PPs has been demonstrated when medical treatments such as mesalazine and azathioprine are utilized. For symptomatic PPs or PPs for which endoscopic criteria alone were unable to exclude malignancy, endoscopic resection with electrocautery is used. Surgical management is reserved for PPs complicated by obstruction or intussusception [14-16].

Most of these cases were managed surgically and only a few cases were treated medically and resulted in regression of the polyp [11-13,17]. The arguments in our case were mainly her presentation of obstructive symptoms and the uncertainty regarding the histology of the deep tissues.

## Conclusions

We report a rare case of giant inflammatory polyposis in long-standing UC presenting with obstructive symptoms mimicking malignancy. A step-up approach with aggressive medical therapy targeting mucosal healing with continuous surveillance would likely prevent these complications.
